# The Mechanism of the Development and Maintenance of Sexual Dimorphism in the Dioecious Mulberry Plant (*Morus alba*)

**DOI:** 10.3390/biology13080622

**Published:** 2024-08-15

**Authors:** Yisu Shi, Michael Ackah, Frank Kwarteng Amoako, Mengdi Zhao, Grace C. van der Puije, Weiguo Zhao

**Affiliations:** 1 Jiangsu Key Laboratory of Sericulture Biology and Biotechnology, School of Biotechnology, Jiangsu University of Science and Technology, Zhenjiang 212100, China; shiyisu1297@126.com (Y.S.); ackahmichael90@gmail.com (M.A.); 2 Key Laboratory of Silkworm and Mulberry Genetic Improvement, Ministry of Agriculture and Rural Affairs, The Sericultural Research Institute, Chinese Academy of Agricultural Sciences, Zhenjiang 212100, China; 3 Institute of Plant Nutrition and Soil Science, Kiel University, Hermann-Rodewald-Straße 2, 24118 Kiel, Germany; kwamekwarteng242@gmail.com; 4 Department of Materials Science and Engineering, Suzhou University of Science and Technology, Suzhou 215011, China; 5 Department of Crop Science, School of Agriculture, College of Agriculture and Natural Sciences, University of Cape Coast, Cape Coast P.O. Box 5007, Ghana; gvanderpuije@ucc.edu.gh

**Keywords:** sex-biased gene expression, sexual dimorphism, sexual selection, pollen competition, haploid selection, artificial selection, species diversity

## Abstract

**Simple Summary:**

This study highlights the importance of intersexual differentiation in dioecious plant speciation and maintenance. Using *Morus alba* plants, we identified 1543 differentially expressed genes (DEGs) between male and female individuals. Notably, 333 genes were exclusively expressed in male inflorescences, while 66 were unique to female inflorescences. Through comparative transcriptomics, 841 genes with significantly higher expression in males were linked to defense-related pathways, and 702 genes with female-biased expression were related to growth and development pathways. Signals of selection were detected in both male- and female-biased genes, indicating their role in evolution. These findings advance our understanding of the molecular basis of intersexual differentiation and shed light on sex evolution in *M. alba* plants.

**Abstract:**

Intersexual differentiation is crucial for the speciation and maintenance of dioecious plants, but the underlying mechanisms, including the genes involved, are still poorly understood. Here, we focused on a typical dioicous plant *Morus alba*, to explore the molecular footprints relevant to sex evolution by revealing the differentially expressed genes (DEGs) between two sexes and the testing signals of selection for these DEGs. From the results, we found a total of 1543 DEGs. Interestingly, 333 and 66 genes expression were detected only in male and female inflorescences, respectively. Using comparative transcriptomics, the expression of 841 genes were found to be significantly higher in male than in female inflorescences and were mainly enriched in defense-related pathways including the biosynthesis of phenylpropanoids, cutin, suberine and waxes. Meanwhile, the expression of 702 genes was female-biased and largely enriched in pathways related to growth and development, such as carbohydrate metabolism, auxin signaling and cellular responses. In addition, 16.7% and 17.6% signals of selection were significantly detected in female- and male-biased genes, respectively, suggesting their non-negligible role in evolution. Our findings expanded the understanding of the molecular basis of intersexual differentiation and contribute to further research on sex evolution in dioecious plants.

## 1. Introduction

Ongoing global change poses a serious threat to the conservation of plant biodiversity and ecosystem functioning [1]. For conservation purposes, it is therefore essential to elucidate how plants respond to changing environments, which requires a comprehensive understanding of their life history [2]. Dioecious plants have many different reproductive traits that have evolved under different selective forces, and the fitness of individuals with different sexes can be influenced by different factors [3,4,5,6]. However, although such intersexual differences call for sex-specific conservation strategies within a single dioecious plant species [3,7], the details of trait differences and the underlying molecular basis and evolutionary trajectories are still poorly understood. Therefore, studies on the gene expression patterns relevant to the traits constituting sexual dimorphism are of great importance for dioecious plants.

Some traits without a direct relation to gamete development but show significant sexual dimorphism are called secondary sexual characteristics [7]. These traits involved in sexual dimorphism in dioecious species mainly include flowering phenology, floral characters (structure, size, and number of flowers in an inflorescence), floral chemical composition such as nutrient content and herbivore-resistant chemicals, and physical defenses [7,8,9]. Furthermore, these traits are thought to influence fitness by affecting pollination, mating, fecundity, and seed viability [10]. Previous studies have shown that in most dioecious plants, males tend to produce more flowers with shorter flower longevity and less synchronous flowering compared to females, and their evolution is likely driven by competition for pollination efficiency and mating competition [11]. In addition, there may be stronger chemical and physical defenses of male flowers in wind-pollinated plants than insect-pollinated species, which may help to ensure sufficient pollen for pollen dispersal [11,12,13]. As for females, their flowers tend to synthesize more auxins to initiate the reproductive program and stimulate seed development, as well as nutrients that can both attract pollinators and facilitate seed growth [14,15]. However, the molecular basis of these differentiated reproductive traits is still unclear. Although some pathways involved in plant metabolism have been identified [7,16,17]. Fortunately, the rapid development of transcriptomics and comparative genomics has shed light on unravelling the molecular mechanisms underlying trait variation by detecting variable expression and signals of positive selection of candidate genes [5,18].

Species in the genus *Morus* are widely distributed and provide an important pool of species for agriculture, but many wild species are threatened [19]. Mulberry (*M. alba*) (2n = 28) is a dioecious wind-pollinated perennial woody plant, cultivated in Asia [20,21]. *M. alba* exhibits apparent sexual dimorphism in flower structure, number and size per inflorescence and flower earlier in males than in females [21,22,23]. This provides an excellent model for screening genes associated with sexual dimorphism. In addition, a high-quality assembly with a genome size of 346.39 Mb has been reported for this species [21], providing a profound basis for transcriptome studies. Furthermore, there are many dioecious and monoecious species in the genus *Morus* [24], so the presence of significant signals of positive selection in the screened genes can be easily tested by interspecific comparisons with closely related monoecious species.

In this study, we used comparative transcriptome analysis to screen differentially expressed genes (DEGs) between two sexes of *M. alba* inflorescences collected from three male and three female trees and performed functional enrichment on selected DEGs relevant to traits of sexual dimorphism, with further evolutionary analysis to uncover their evolutionary history. We aim to answer two scientific questions: (1) whether DEGs in male and female inflorescences are involved in regulating the development of sex-specific traits, and (2) whether these genes are under positive selection. Our results will provide theoretical evidence and elucidate the molecular basis of the origin and maintenance of sexual dimorphism in plants and will aid future conservation efforts.

## 2. Materials and Methods

### 2.1. Plant Materials

**Figure 1:**
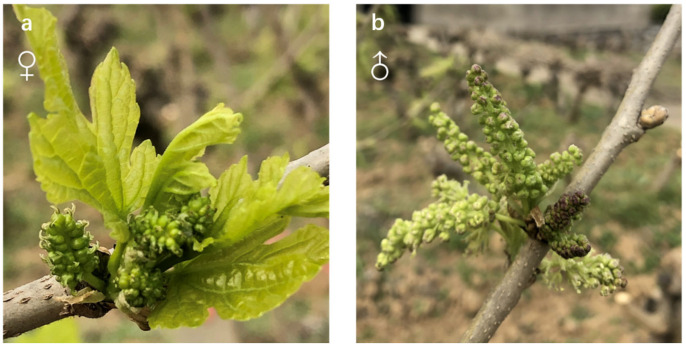
Physical appearance of collected adult *Morus alba* catkins. (**a**) Female catkins with protruding pistillate flowers. (**b**) Male catkins with protruding staminate flowers.

The flower buds were collected from the trees of two *M. alba* purified breeding lines, three BaiTiao (BT), which produces only male flowers, and three Y20 which produces only female flowers [25]. These breeds are grown at the Sericulture Research Institute of the Chinese Academy of Agricultural Sciences in Zhenjiang, Jiangsu Province, China (N 32°11′45.80″, E 119°23′45.80″). Maintenance conditions for the *M. alba* tree are as follows: minimum and maximum temperatures (9–20 ℃), relative humidity (40–60%), irrigation (rainfall), day length (12 h), and light intensity (15,000 lx). Additionally, information about the appearance conditions of the trees includes age (5 year), diameter (5 cm), and height (2 m). BT and Y20 have characteristics such as larger inflorescences, a greater number of flowers, and wide adaptability and are typical cultivars of *M. alba*. Therefore, these two purified lines were used as the study materials to compare the differences in gene expression between male and female inflorescences of *M. alba*. Male and female inflorescence (1.3 m from the ground) samples were collected (that is, male inflorescence from BT purified lines and female inflorescence from the purified Y20 lines). The samples were a mixture of different inflorescences from the same plant, and six trees (3 from BT and Y20 each) were selected for sampling, representing three biological replicates. In all, a total of six catkins were sampled. Inflorescence sampling was conducted from 9:00 p.m. to 11:00 p.m. Only fully developed adult catkins (0.8–1.5 cm long and 0.3–0.5 cm in diameter) were collected, when the catkins were fully expanded, but before the flowers had opened (Figure 1). Whole catkins were cut and immediately frozen in liquid nitrogen for RNA extraction.

### 2.2. RNA Extraction and Illumina Sequencing

Using the RNAiso Plus reagent (Takara, Shanghai, China), reverse transcription and total RNA extraction were performed on each sampled catkin in accordance with the manufacturer’s instructions. Using an Agilent 2100 Bioanalyzer (Agilent Technologies, Palo Alto, CA, USA) and NanoDrop 1000 spectrophotometer (IMPLEN, Westlake Village, CA, USA), the quantity and purity of total RNA were assessed. Furthermore, 1 μg of total RNA per sample was used to construct an RNA sequence library using the NEBNext^®^UltraTM RNA Library Prep Kit for Illumina (NEB, Ipswich, MA, USA) according to the manufacturer’s specifications. Subsequently, transcriptome sequencing was conducted using an Illumina Novaseq 2500 platform (San Diego, CA, USA), which produced paired-end reads of 150 bp. 

### 2.3. Quality Control of Transcriptome Data and Expression Analysis

To obtain clean RNA-seq data, the raw RNA-seq reads from each sample were processed using in-house perl scripts to trim adapters and low-quality bases from the ends of the reads, with further filtering of reads containing poly-N. The clean, high-quality reads for each sample were then mapped to the *M. notabilis* reference genome [20] using Hisat2 v2.0.5, and the mapped reads were assembled using StringTie (v1.3.3b) for novel transcript prediction. Fragments per kilobase million (fPKM) was used to estimate gene expression levels from each gene according to the number of reads mapped to that gene region using featureCounts v1.5.0-p3. Differential expression analysis was performed to detect the significantly differentially expressed genes (DEGs) between the male and female catkins using the DESeq2 R package (v1.16.1) [26]. Furthermore, *p*-values were adjusted based on the false discovery rate (FDR) using the Benjamini and Hochberg approach. Genes with a fold change difference in expression (|log2(FoldChange)| > 0) or (|log2(FoldChange)| < 0) and adjusted *p*-values (*p*-adj) < 0.05 were considered as DEGs. Among these DEGs, the value of (|log2(FoldChange)| > 0) was defined as male-biased genes and (|log2(FoldChange)| < 0) as female-biased genes. The extremes were considered as genes expressed only in females and were defined as female-limited genes, and the opposite was defined as male-limited genes. Gene ontology (GO) enrichment analysis was performed on the DEGs using the ClusterProfiler R package [27]. GO terms with padj < 0.05 were considered significantly enriched by DEGs. The ClusterProfiler R package was also used to perform DEG enrichment statistics in the KEGG pathway [27]. Pop_tri_v3 (*Populus trichocarpa*) was used as a reference (https://plants.ensembl.org/Populus_trichocarpa/Info/Index, accessed on 15 October 2023).

### 2.4. RT-qPCR Verification

Based on the KEGG and GO enrichment results of the DEGs, 10 female-biased/-limited expression genes enriched in growth and development pathways and 10 male-biased/-limited expression genes enriched in defense-related pathways (Table S1) were randomly selected for RT-qPCR analysis. The RNA samples of flower buds used for RT-qPCR were taken from the same individuals as the samples used for transcriptome sequencing. A total of 20 pairs of gene-specific primers were constructed based on their sequences in the reference genome utilizing Primer 6 software (Table S1), and cDNA synthesis was performed using M-MLV reverse transcriptase (RTase) (Takara, Beijing, China), with 1 µg RNA samples as the template. The cDNA solution was diluted 5-fold, and 1 µL of cDNA was used as the template to perform gene validation using the SYBR Green RT-PCR protocol (Roche, Indianapolis, IN, USA). The reaction system contained SYBR qPCR 2×Taq Mix (10 µL), forward primer (1 µL), reverse primer (1 µL), ddH2O (7 µL) and cDNA (1 µL), and the β-actin gene (as a normalization control); the primer of the β-actin gene is shown in Table S1. All reactions were performed in three biological replicates using three templates. The procedure for the RT-qPCR was as follows: 95 °C for 10 min, followed by a cycle program (denaturation: 95 °C for 10 s, annealing: 50 °C for 10 s, and extension: 70 °C for 10 s) for 45 times. The mean relative expression of each gene was normalized to the reference gene, β-actin, and calculated using the 2^−ΔΔCt^ method [28].

### 2.5. Analysis of Adaptive Evolution of Sex-Biased and Unbiased Genes

To test whether the sex-biased genes expressed only in males or females and the sex-biased DEGs had undergone significant positive selection, we used the evolutionary ratio (dN/dS) for all *M. alba* genes using paml v4.9 [29]. For each *M. alba* gene, the nucleotide sequences were translated into protein sequences, and their coding sequences were searched in TBtools v11.0.2 [30]. As *M. alba* shares a common ancestor with *M. notabilis* and diverged with *Prunus persica,* which is a hermaphrodite [21], we compared the coding sequences from our *M. alba* transcriptome data with the homologous genes in the *M. notabilis* genome [20]. Moreover, the sequences from the *P. persica* genome were used as an outgroup. Based on the bit score, e-value, and local alignment length (Supplementary Materials, R script), we used blastn 2.12.0 to identify the optimal multidirectional matching homologs to *M. alba* from *M. notabilis* v2.0 [20] and *P. persica* (Peach v1.0) [31] genome annotation data. The coding and protein sequences of the three species alignments were extracted and compared using ParaAT v1.0 [32]. We estimated the dN/dS values for the *M. alba* and *M. notabilis* lineages with codeml (PAML v4.9) [29], using a model with two or more dN/dS ratios for branches, runmode as user tree, and ndata as 13,532, and other parameters were set as the default [33]. We excluded 4980 alignments from the analysis due to unrealistically high divergence (S × dS + N × dN > 15% of alignment) [7].

In addition, we conducted a comparison of the dN/dS values for the evolutionary lineage leading to *M. alba* and *M. notabilis*, specifically focusing on genes that exhibit sex-biased or sex-limited expression patterns, as well as genes that do not display such biases. Kruskal–Wallis tests were used to compare the evolutionary ratio (dN/dS) between the sex-biased and unbiased genes, which is also used in Sanderson et al. (2019). Initially, a non-parametric Kruskal–Wallis test was employed to assess the differences in median values of dN/dS between sex-biased genes and a randomly selected unbiased genes (Kruskal.test and dunn.test in R to perform the Kruskal–Wallis test and post hoc tests respectively). To ascertain the rate of neutral evolution for these genes, a comparative analysis was conducted on the median dS values of sex-biased genes and a randomly selected set of 3000 unbiased genes [7]. Additionally, we conducted a comparison of the 95% quantile values of dN/dS across three categories in order to assess whether genes exhibiting sex-biased or sex-limited characteristics were more prone to displaying extreme values compared to the distribution of 95% quantile values derived from 5000 bootstrap samples of unbiased genes. This analysis was performed using the boot package in the R programming language (https://CRAN.R-project.org/package=boot, accessed on 12 August 2024). To explore the pathways and functions of positively selected DEGs, KEGG and GO enrichment analysis for the positively selected DEGs were performed using the clusterProfiler R package (*p*-adj < 0.05).

## 3. Results

### 3.1. Sequencing Quality Control and Reference Genome Mapping

Six libraries were subjected to Illumina sequencing utilizing samples from male and female flower buds. Each sample was prepared in three repetitions. A combined sum of 305,642,716 raw sequencing reads were acquired from the flower buds of BT (male) and Y20 (female). After trimming adapter and poor-quality sequences, 294,445,006 clean reads were obtained. Among these reads, 139,671,832 (95.6%) were clean reads from male flower buds and 154,773,174 (97.0%) were clean reads from female flower buds. For all the libraries, the average Q30 was more than 92% and the GC content was more than 40% (Table 1). Furthermore, more than 64.5% of the clean reads were mapped to the reference genome (*M. notabilis*). A total of 21,155 unigenes were obtained from the assembly, with the longest gene being 16,567 bp and the shortest having 72 bp.

### 3.2. Differentially Expressed Genes between Male and Female Flower Buds 

**Figure 2:**
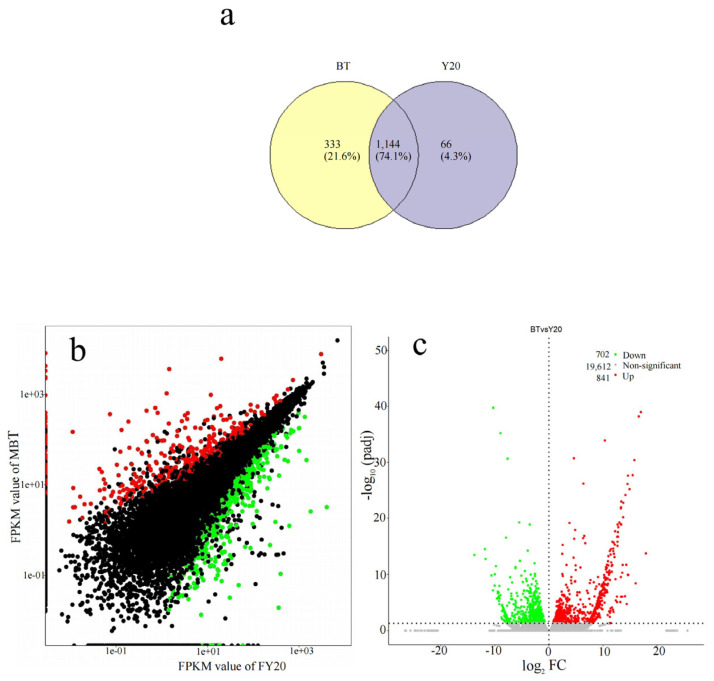
Comparisons of gene expression between male and female flower buds of *M. alba*. (**a**) Shared differentially expressed genes between male and female flower buds. (**b**) Gene expression comparison between male and female flower buds. (**c**) Volcano plot on sex-biased genes that were expressed significantly different in male and female flower buds.

Following the transcriptome assembly and annotation, differential expression analysis was performed. Surprisingly, a total of 1,543 genes were differentially expressed in male and female flower buds based on the criteria stated in Section 2.3. Out of these DEGs, 702 genes were significant in female flower buds and 841 genes were significant in male flower buds (Figure 2). A total of 19,612 genes exhibited a transcript abundance difference that did not meet the criteria (Figure 2c). These findings indicate the presence of biologically significant levels of gene expression dimorphism. Genes that were expressed exclusively in one sex type were defined as sex-limiting genes (DEGs showing some expression in one sex type but absolutely zero counts in the other). A total of 399 genes exhibited expression that was sex-limited. Out of the total, 333 genes (83.5%) were exclusively expressed in male flowers, while 66 genes (16.5%) were exclusively expressed in female flowers, suggesting that the proportion of male-biased genes was higher than that of female-biased genes. The number of genes exhibiting male bias was marginally higher compared to those exhibiting female bias.

**Figure 3:**
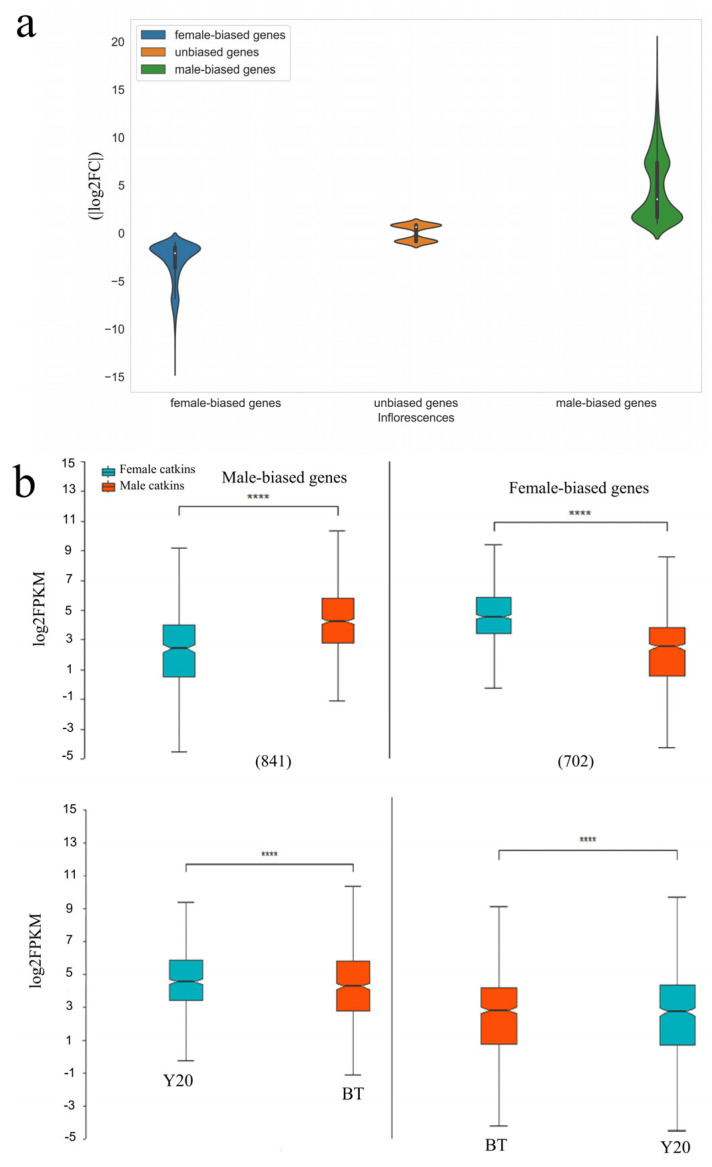
Sex-biased gene expression in *M. alba*. (**a**) The proportion and range of DEGs and unbiased genes in *M. alba* catkins. (**b**) The upper-half shows the comparison of male and female catkin differentially expression genes (DEGs) at different sex bias fold change thresholds for all evaluated catkin male-biased and female-biased genes. The numbers in the brackets represent the number of DEGs in each catkin category. The lower-half shows the average male and female catkin expression of all genes at different sex bias fold change thresholds for all the evaluated catkin male-biased and female-biased genes. Significant differences between male and female expression based on Wilcoxon rank sum tests are denoted by asterisk at alpha level (*p* < 0.0001). The sex-biased genes mentioned here include sex-limited genes. BT and Y20 are the male and female catkins.

Additionally, the extent of differential expression, as measured by (|log2FC|), was significantly higher for genes with male bias compared to those with female bias (Figure 3a, Wilcoxon rank sum test *p* < 0.01, R 4.1.0). The mean expression levels of male-biased genes were found to be considerably lower compared to the average expression levels of female-biased genes (Figure 3b; Wilcoxon rank sum test *p* < 0.01, R 4.1.0), although female expression for male-biased genes was significantly higher than male expression for female-biased genes (Figure 3b, Wilcoxon rank sum test *p* < 0.01, R 4.1.0). The findings of this analysis indicate that genes with a bias towards females may originate from heightened expression in female inflorescences and reduced expression in male inflorescences. Additionally, it is seen that the bias towards males predominantly stems from the upregulation of female expression.

### 3.3. Functional Annotation Associated with DEGs 

**Figure 4:**
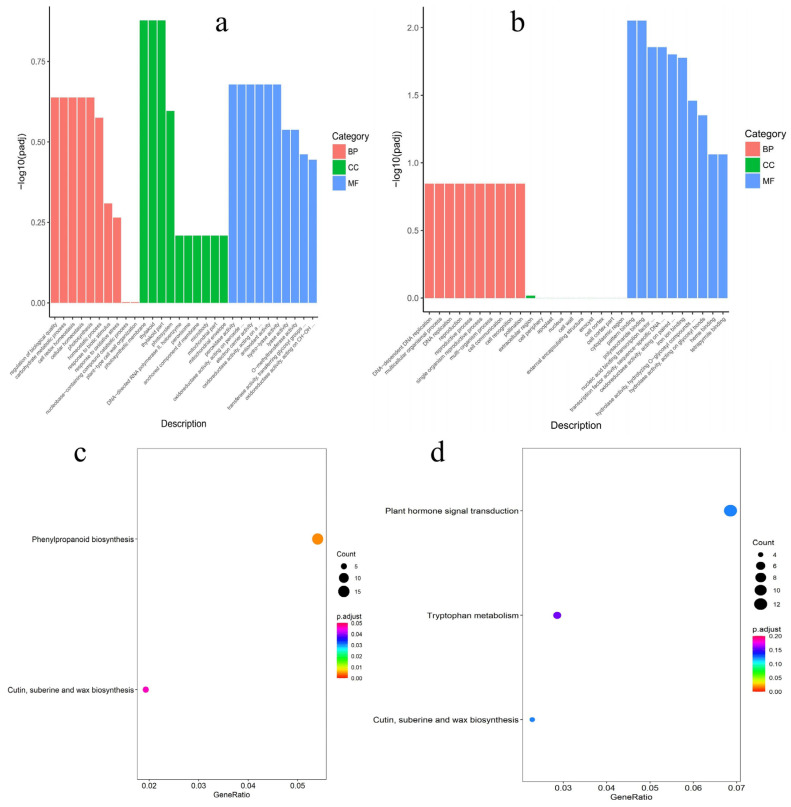
Functional analysis of the DEGs in *M. alba* male and female catkins. (**a**) GO enrichment terms of male-biased expression genes. (**b**) GO enrichment terms of female-biased expression genes. (**c**) KEGG-enriched differential expression genes of male-biased expression genes. (**d**) KEGG-enriched differential expression genes of female-biased expression genes. BP; biological process. CC; cellular component. MF; molecular function.

**Figure 5:**
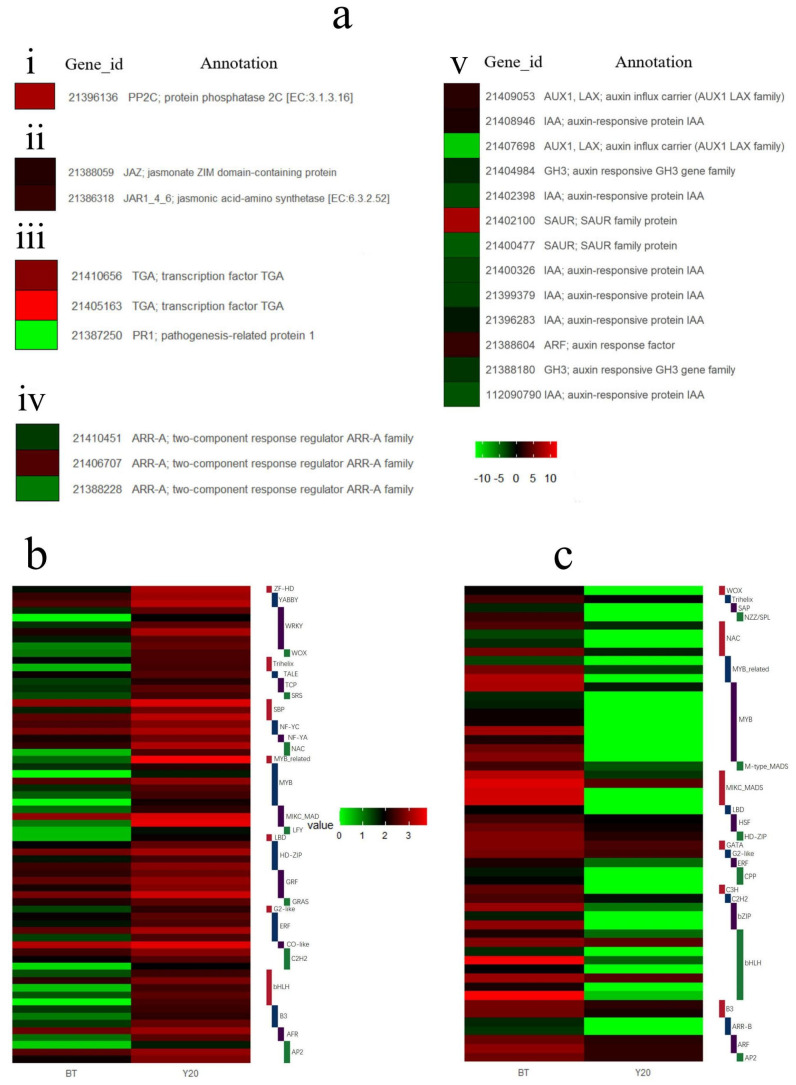
A heat map analysis of the DEGs in *M. alba* male and female catkins. (**a**) A heat map diagram of the DEGs between male and female flower buds of *M. alba* in the phytohormone signaling pathways: abscisic acid (i), jasmonic acid (ii), salicylic acid (iii), cytokinin (iv), and auxin (v). (**b**) The transcription factor (TF) families differentially expressed in male-biased genes. (**c**) The TF families differentially expressed in male-biased and female-biased genes. The color scale represents the log_10_-transformed FPKM value. The sex-biased genes mentioned here include sex-limited genes. The male-biased genes are upregulated and female-biased genes are downregulated based on the figure legend.

To determine whether these DEGs are associated with the development of sex-specific traits, GO and KEGG analysis was used to identify patterns of enrichment between the DEGs in male and female flower buds. According to the GO enrichment results, the most female-biased genes were annotated in nucleic acid binding transcription factor activity (GO:0003700), transcription factor activity, and sequence-specific DNA binding (GO:0001071) (Figure 4a,b). The most male-biased genes were annotated for carbohydrate metabolism processes (GO:0005975). To further understand the enrichment pathways of male-/female-biased genes, these DEGs were analyzed by KEGG pathway analysis. Male-biased genes were enriched in 111 KEGG pathways and significantly enriched in the phenylpropanoid biosynthesis pathway (pop00940). Female-biased genes were assigned to 101 KEGG pathways and were mainly enriched in the plant hormone signal pathway (pop04075) (Figure 4c,d). The role of plant hormones in plant sex determination has been demonstrated in many studies [34,35]. According to the result of KEGG enrichment analysis, 22 DEGs (10 female-biased and 12 male-biased) were enriched in plant hormone signaling, including auxin, cytokinin, salicylic acid, jasmonic acid, and abscisic acid signaling pathways (Figure 5a). 

Interestingly, the male-biased genes were mainly annotated in the abscisic acid and jasmonic acid signaling pathways, whereas the female-biased genes were mainly annotated in the cytokinin and auxin signaling pathways. The screened DEGs contained 88 transcription factors (TFs) that could be successfully annotated for the KEGG pathway, including 33 female-biased TFs and 55 male-biased TFs.

Among them, the female-biased/-limited TFs were annotated for 29 KEGG pathways, which were related to growth and development. They include metabolic pathways (pop01100), biosynthesis of secondary metabolites (pop01110), starch and sucrose metabolism (pop00500), and so on. However, the male-biased/-limited TFs were annotated for 66 KEGG pathways, including metabolic pathways (pop01100), biosynthesis of secondary metabolites (pop01110), glycerophospholipid metabolism (pop00564), plant hormone signal transduction (pop04075), glycolysis/Gluconeogenesis (pop00010), glycerolipid metabolism (pop00561), and fatty acid metabolism (pop01212). TFs’ regulation of gene expression and the expression profiles of TF genes between male and female flower buds are shown as a heat map (Figure 5b,c). 

Combining the results of KEGG and GO enrichment analysis, it was found that many metabolic processes in inflorescences exhibit sexual dimorphism in gene expression. Floral transcripts in the phenylpropanoid biosynthesis pathway, which produces important defense compounds, showed an increased abundance in male inflorescences compared to female inflorescences (14 male-biased and 2 male-limited genes among a total of 25, *p* = 1.7 × 10^−3^; Table S3). The pyruvate pathway related to the production of volatiles against herbivores was also dominated by male-biased/-limited genes (11 of 13, including 3 male-limited genes, *p* = 1.3 × 10^−3^; Table S3). In contrast, transcript abundance was higher in female inflorescences compared to male inflorescences for plant hormone signaling (21 out of 35, including 10 female-limited genes, *p* = 8.5 × 10^−4^) (Table S3) and plant–pathogen interaction (11 out of 17, including 5 female-limited genes, *p* = 3.2 × 10^−4^, Table S3). These suggest that female inflorescences may invest more in growth and less in defense, making them more susceptible to plant pathogens, whereas male inflorescences may invest more in protection from herbivores. 

### 3.4. Verification of DEGs via Real-Time Quantitative PCR

**Figure 6:**
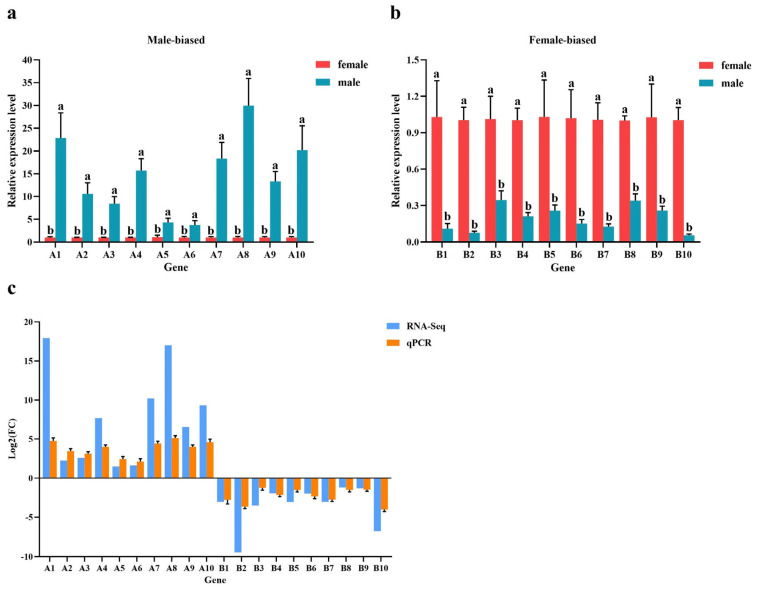
RT-qPCR validation of differentially expressed genes between male and female *M. alba* flower buds, including 9 male-biased genes and 1 male-limited gene (**a**) and 9 female-biased genes and 1 female-limited gene (**b**). (**c**) Comparation of gene expression results of qPCR and RNA-seq. A1; stamen-specific protein FIL1 (male-limited gene). A2; probable aminotransferase TAT2. A3 pollen-specific leucine-rich repeat extension-like protein3. A4; pollen-receptor-like kinase1. A5; vesicle-associated. A6; protein eceriferum 12C. A7; PHD finger protein male sterility1. A8; plant UBX domain-containing protein 2. A9; endoglucanase 2C. A10; transcription factor DYT1. B1; auxin-responsive protein IAA32. B2; auxin transporter-like protein 2 (female-limited gene). B3 auxin-induced protein AUX22. B4: auxin efflux carrier component 3. B5; auxin-responsive protein IAA4. B6; alcohol dehydrogenase-like 2C. B7; protein PIN-LIKES 3. B8; auxin response factor 42C. B9; nudix hydrolase 2C. B10; transcriptional regulator SUPERMAN. Error bars represent mean ± SD of three replicates of relative expression of each gene. Bars with different letters indicate significant differences between expression levels for each gene (*p* ≤ 0.05) based on Duncan’s multiple range test.

To confirm the reliability of the RNA-seq data, 20 female- or male-biased unigenes annotated for defense-related pathways and growth and development pathways, respectively, were randomly selected for performing real-time quantitative PCR, including 10 (9 + 1) male-biased genes and 10 (9 + 1) female-biased genes (Figure 6; Table 2). Although the expression values were different between RT-qPCR and RNA-seq, the expression patterns of the genes were consistent with RNA-seq. The male-biased genes were significantly upregulated in males and downregulated in females and vice versa (*p* ≤ 0.05).

### 3.5. Evolution of Sex-Limited and Sex-Biased Expressed Genes

**Figure 7:**
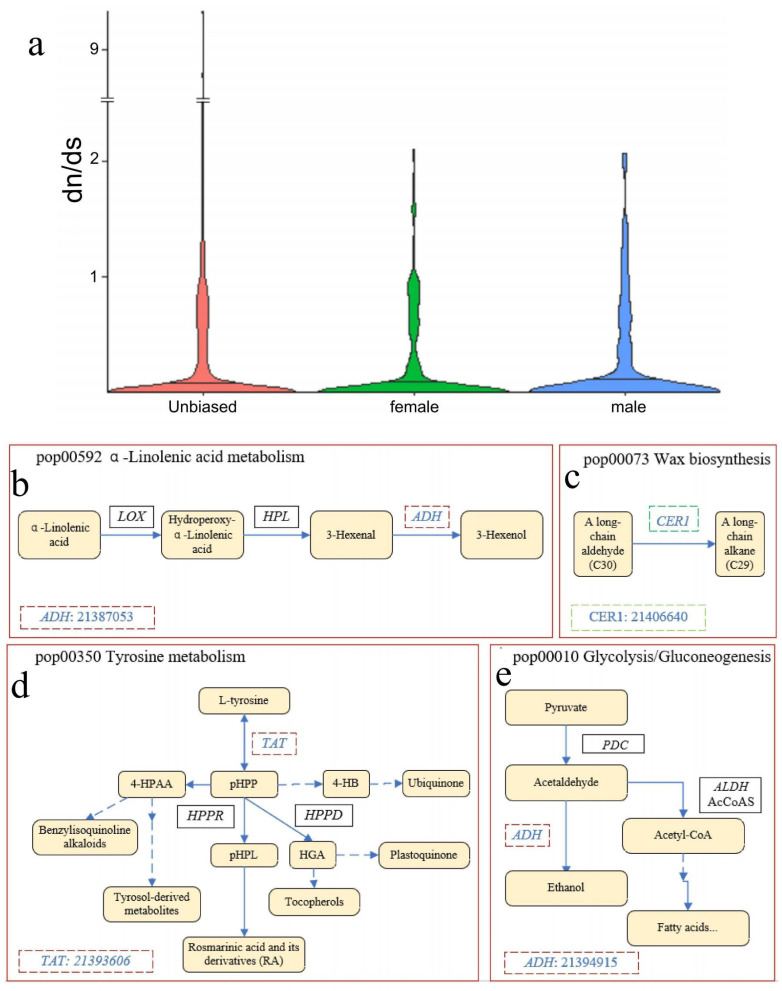
(**a**) Distribution of dN/dS for genes biased in expression in female and (green) or male (blue) in *M. alba*, compared with genes randomly chosen from unbiased genes. Center line represents median value of dN/dS distribution. (**b**–**e**) Relevant biological processes and possible functions involved in male and female DEGs subjected to positive selection. *ADH*; genes encoding alcohol dehydrogenase; *CER1*; ECERIFERUM1 gene predicted to encode enzyme involved in alkane biosynthesis; *TAT*; genes encoding tyrosine aminotransferase. Genes in red dashed boxes are male-biased genes, and genes in green dashed boxes are female-biased genes. Corresponding gene ID is in lower-left corner.

To investigate those genes (i.e., sex-limited and sex-biased) associated with sex-specific traits under selection, we calculated the dN/dS of these genes. The median dN/dS of male-biased genes was significantly higher than that of female-biased genes and randomly selected unbiased genes (Figure 7a; Kruskal–Wallis test, *p*-value = 0.03447, df = 2, Chi-squared = 6.7356). The distribution of dS values showed the completely opposite pattern to that observed for dN/dS values. The dS values of sex-biased genes were slightly higher than those of random samples of unbiased genes (Kruskal–Wallis test, *p*-value = 0.7956, df = 2, Chi-squared = 0.4757). 

To further discuss the situation of extreme values of dN/dS in sex-biased genes and unbiased genes, we calculated and compared the distribution of the upper 95% quantiles of 5000 random bootstrap samples of unbiased genes and the upper 95% quantile of dN/dS of sex-biased genes. The results show that the upper 95% quantiles for the sex-biased genes (9.8876) exceeded the quantiles from the bootstrap sampling (3.721–5.039), suggesting that sex-biased genes were more likely to enrich the maximum dN/dS than unbiased genes. Remarkably, a total of 18 DEGs (5 female-biased genes (4 + 1) and 13 male-biased genes (10 + 3)) under positive selection (dN/dS > 1) were screened (Table S2), and only 6 DEGs were enriched in 18 KEGG pathways (Table S3). These included one female-biased gene enriched in cutin, suberine, and the wax biosynthetic pathway (pop00073) and four male-biased genes mainly enriched in the synthesis and metabolism of multiple amino acids and secondary metabolites, including glycolysis/gluconeogenesis (pop00010), tyrosine metabolism (pop00350), α-Linolenic acid metabolism (pop00592), and phenylpropanoid biosynthesis (pop00960) (Figure 7b). 

Comparing the results of the KEGG enrichment analysis with these evolutionary results, we found that the male-biased expressed genes under positive selection (dN/dS > 1) are mostly enriched in the synthesis and metabolism of several amino acids and secondary metabolites (4 unigenes). In the present research, two positively selected genes (gene ID: 21394915 and 21387503) were involved in the biosynthesis of alcohol dehydrogenase (Devani et al.) [34] homologous genes and were expressed with a male bias (Figure 7). 

## 4. Discussion

The present study examines the differential gene expression in the inflorescences of female and male individuals of *M. alba*, revealing notable distinctions between the two sexes. Results from other studies suggest that sexual dimorphism is mainly reflected in floral tissues [7,36,37]. Therefore, we believe this sex-biased gene expression in inflorescences might contribute to the morphological differences between male and female inflorescences. We also found that most of the male-biased/limited expression genes were involved in the synthesis of secondary metabolites, but most of the female-biased genes were involved in the regulation of growth-related metabolic processes. Some of the sexually biased genes in *M. alba* inflorescences exhibited molecular evolutionary signals. Furthermore, a large number of genes involved in the formation of phenotypic differences between male and female inflorescences were found to be positively selected during natural selection. These results bring us closer to answering the long-standing question of whether DEGs in male and female inflorescences are involved in regulating the formation of sex-specific traits and what evolutionary pressures have been exerted on these genes.

Comparative analysis of the transcriptome profile between male and female inflorescences can effectively narrow down the range of candidate genes for sex differentiation. Numerous studies have been conducted to examine the differential expression of genes related to sexual dimorphism in dioecious plant species. According to Zemp et al. (2016) [6], a study conducted on *Silene latifolia* revealed that approximately 17% of genes exhibited differential expression in flowers. Similarly, Darolti et al. (2018) [3] found that in *Salix viminalis*, approximately 43% of genes displayed differential expression, specifically in catkins. In *Populus. balsamifera*, 36% of genes expressed in catkins show a sex bias [7]. In the current study of *M. alba*, we found that only 7.3% of genes are differentially expressed in inflorescences, of which 45.5% are female-biased and 54.5% are male-biased. This result is partially consistent with findings from a previous study where it was found that the number of male- and female-biased genes was approximately equal in *Salix viminalis* [3,7]. Nevertheless, the observed sex-biased gene expression pattern in this particular species stands in contrast to that of two other dioecious species, namely, *Silene latifolia* and *Asparagus officinalis*, where a greater proportion of genes demonstrated male-biased expression in flowers [6,7,38]. The more male-biased expression of genes in these studies can be explained by the fact that insect-pollinating flowers need to expend energy to produce more pollinator-attracting traits than wind-pollinated flowers and that these traits are usually strongly influenced by sexual selection. Alternatively, the equal amount of male- and female-biased expression genes in *M. alba* inflorescences may reflect the lower energetic demands of attracting pollinators to compete for mating. Therefore, it is plausible that variations in gene expression between male and female inflorescences may contribute to the distinct physical attributes of each and ultimately result in sex differentiation.

Functional annotation revealed that these sex-biased expression genes were largely enriched in phenylpropanoid biosynthesis, cutin, suberine, and wax biosynthesis and fatty acid elongation pathways. Thus, male-biased/-limited expression genes were involved in the production of some secondary metabolites such as defenses, regulators, and primary metabolites. On the other hand, female-biased/-limited expression genes mainly enriched in pathways related to growth and reproduction. We observed an expression dimorphism in plant–pathogen interaction pathways, suggesting that female inflorescences are more susceptible to plant pathogens. The gene *CER1,* which is involved in regulating the conversion of C30 aldehydes to C29 alkanes in the cutin, suberine, and wax biosynthesis pathways [39], showed female-biased expression in our study. The overexpression of *CER1* was also shown to increase susceptibility to bacterial and fungal pathogens [39]. In Arabidopsis, this enzyme is thought to be involved in catalyzing the conversion of C30 aldehydes to C29 alkanes during the biosynthesis of cuticular waxes, which alters the permeability of the cuticle and affects the susceptibility of the epidermis to bacterial and fungi [39]. This may be related to the need for females to invest more in the care of the next generation. This also may indicate that in mulberry, males invest more in the protection of natural enemies than females do during development. Thus, females and males may have been subject to different selective pressures during their evolution, which may explain the sexual differentiation of *M. alba*. Another positively selected male-biased gene (21393606) belongs to the tyrosine aminotransferase [31] genes, which was involved in the tyrosine metabolism pathway (Figure 7d). This suggests that male inflorescences may be more resistant to biotic stresses than female inflorescences. In contrast, the *ADH* gene, which is involved in aerobic fermentation and the production of characteristic scents that act to protect against herbivores [40], showed male-biased expression, suggesting that males, unlike females, invest more in defense. The probable function of *ADH* has been extended to include roles in aerobic fermentation in pollen grains (buffering the system, regulating carbon flow to protect the cell from acetaldehyde toxicity) in the production of characteristic scents such as 3-Hexenol, that act as protection against herbivores [40] (Figure 7b,e). While the female-biased expressed genes by positive selection (dN/dS > 1) were mainly enriched in cutin, suberine, and wax biosynthesis pathways (2 unigenes), one of them belongs to the *CER1* gene family (21406640), encoding an aldehyde decarboxylase involved in the biosynthesis process of long-chain alkanes in this study (Figure 7c). 

TAT homologs exhibit a broad distribution across plant species and play a significant role in the synthesis of a wide range of structurally diverse compounds in plants. These molecules include tocopherols, ubiquinone, plastoquinone, betalains, salidroside, benzylisoquinoline alkaloids, and rosmarinic acid, among others (Figure 7d). They are involved in the synthesis of defense compounds against a wide range of herbivores [17]. In the majority of plant species, tyrosine aminotransferase (TAT; EC 2.6.1.5) catalyzes the reversible transamination of tyrosine to form 4-hydroxyphenylpyruvic acid (phPP), a precursor for the production of downstream specialized metabolites such as tocopherols, plastoquinone, and ubiquinone, which assist plants in adapting to and thriving in different environments [41]. In our study, the genes encoding *TAT* were expressed in a male-biased manner, suggesting that male inflorescences may be subject to stronger environmental selection than female inflorescences. In conclusion, such sexual dimorphism due to sex-biased gene expression may act as a selective pressure for sexual specialization. We expected that in *M. alba*, the effect of natural selection on the different phenotypes between male and female inflorescences would have altered gene frequencies and caused adaptive evolution, which could account for sexual dimorphism. However, for *M. alba*, the average value of dN/dS was approximately between 0.15 and 0.25, which means that at least 75–85% of nonsynonymous mutations are deleterious. Male-biased genes in *M. alba* showed increased molecular evolutionary signals (higher dN/dS) compared to female-biased genes and unbiased genes, suggesting that male-biased genes may contain more favorable mutations regardless of population size. In the dioecious *P. balsamifera*, male-biased and female-biased genes had lower dN/dS than unbiased genes, suggesting that sexually biased genes with fundamental functions suffer from greater purifying selection to eliminate deleterious alleles [7]. Again, this can be used to explain the results of our study in the sense that male-biased expression genes with defense functions are favored by natural selection.

Our results are generally consistent with previous reports that there are a large number of sex-biased expression genes in the inflorescence of dioecious plants. The sex-biased expression genes are involved in regulating the formation of sex-specific traits that differentiate developmental patterns between the two sexes, which may explain the occurrence of intersexual differentiation in dioecious plants. In *M. alba*, we observe that male-biased expressed genes are mainly enriched in the synthesis pathways of some secondary metabolites such as defenses, regulators, and primary metabolites, while female-biased expressed genes are mainly enriched in the regulation of metabolic processes related to growth. The enrichment results remain unchanged when only those sex-biased genes under positive selection are considered. These basic findings suggest that natural selection stresses reproductive traits in female and male inflorescences, resulting in sexually dimorphic gene expression, which may be the molecular basis for sexual differentiation in dioecious plants. Unfortunately, we cannot identify the critical genes that regulate female or male flowering in *M. alba*. And our results lack functional validation of candidate genes at this point. Future work will focus on screening for candidate genes that determine which flowers (male or female) are produced by *M. alba*, based on the results of functional enrichment and evolutionary dynamics analysis, and analyzing the functions of these genes. 

## 5. Conclusions

The comparative transcriptome analysis reveals that male and female catkins express a remarkable number of genes with sex-specific expression. The majority of sex-biased genes in catkins are associated with secondary sexual characteristics other than the androecium or gynoecium’s primary sexual characteristics, such as energetic demands and defense against herbivore. The male-biased genes are involved in defense-related physiological processes, in contrast to the female-biased genes, which are mainly involved in the regulation of metabolic processes related to growth and development. The results remain unchanged when only the sex-biased genes under positive selection are considered. This suggests that sexual dimorphism in gene expression may be due to differences in energy and resource allocation between the two sexes, and that such differences are influenced by natural selection and may lead to the maintenance of dioecy in *M. alba*. Unfortunately, we cannot identify the critical genes that regulate female or male flowering in *M. alba*. And our results did not investigate function validation of candidate genes at this time, which will be investigated in the future works. However, our findings have broadened our understanding of the molecular basis of intersexual differentiation and contributed to further research into the evolution of sex in *M. alba* plants.

## Figures and Tables

**Table 1:** Summary of quality preprocessing of RNA sequencing data.

Sample ID	Raw Reads	Clean Reads	Clean Bases	Error Rate	Q30	GC%	Mapping Ratio
MBT-1	52,365,500	50,926,984	7.64 G	0.03	93.12	44.31	64.6
MBT-2	44,385,448	41,784,712	6.27 G	0.03	93.55	42.81	58.4
MBT-3	49,325,358	46,960,136	7.04 G	0.03	92.19	40.73	72.8
FY20-1	56,900,778	54,807,454	8.22 G	0.03	92.27	44.23	55.8
FY20-2	48,518,050	47,476,836	7.12 G	0.03	93.46	44.66	66.9
FY20-3	54,147,582	52,488,884	7.87 G	0.03	92.38	43.44	68.5

MBT and FY20: Male and female mulberry (*Morus alba*) flower buds used in RNA sequencing. MBT-1, MBT-2, and MBT-3 and FY20-1, FY20-2, and FY20-3: three replicates of male and female mulberry (*M. alba*) flower buds, respectively.

**Table 2:** Summary of differential expression genes in male and female flowers used in RT-qPCR verification.

Gene ID	Gene Description	Gene ID	Gene Description
Female-Biased		Male-Biased	
21400326	auxin-responsive protein IAA32	21398828	stamen-specific protein FIL 1
21407698	auxin transporter-like protein 2	21393606	probable aminotransferase TAT2
21402398	auxin-induced protein AUX22	21396084	pollen-specific leucine-rich repeat extension-like protein 3
21400361	auxin efflux carrier component 3	21401585	pollen receptor-like kinase 1
21399379	auxin-responsive protein IAA4	21391819	vesicle-associated protein 2-2
21406640	protein eceriferum 2C transcript variant X2	21394915	alcohol dehydrogenase-like 2C transcript variant X1
21390461	protein PIN-LIKES	21387539	PHD finger protein male sterility1
21400414	transcriptional regulator SUPERMAN	21408700	transcription factor DYT1
21397544	auxin response factor 2C transcript variant X1	21387503	plant UBX domain-containing protein 2
21387910	nudix hydrolase 2C transcript variant X2	21390125	endoglucanase 2C transcript variant X1

## Data Availability

The data used are described in this study. Further inquiries can be made to the corresponding author(s) on reasonable request. Raw RNA-seq data have been submitted to NCBI SRA with the accession number (PRJNA1144468) which will be available upon the release date.

## Supplementary Materials

The following supporting information can be downloaded at https://www.mdpi.com/article/10.3390/biology13080622/s1. Excel file; Tables S1–S3. Notepad file; R script.
